# Effects of Different Cultivation Parameters on the Production of Surfactin Variants by a *Bacillus subtilis* Strain

**DOI:** 10.3390/molecules23102675

**Published:** 2018-10-18

**Authors:** Attila Bartal, Aruna Vigneshwari, Bettina Bóka, Mónika Vörös, István Takács, László Kredics, László Manczinger, Mónika Varga, Csaba Vágvölgyi, András Szekeres

**Affiliations:** 1 Department of Microbiology, Faculty of Science and Informatics, University of Szeged, H-6726 Szeged, Közép fasor 52., Hungary; bartaloszi@gmail.com (A.B.); arunabio2011@gmail.com (A.V.); boka.tina@gmail.com (B.B.); voros.monesz@gmail.com (M.V.); taki.biotech@gmail.com (I.T.); kredics@bio.u-szeged.hu (L.K.); manczinger.laszlo@invitel.hu (L.M.); varga.j.monika@gmail.com (M.V.); mucor1959@gmail.com (C.V.); 2 Doctoral School in Biology, Faculty of Science and Informatics, University of Szeged, H-6726 Szeged, Hungary

**Keywords:** *Bacillus subtilis*, cyclic lipopeptides, HPLC-ESI-MS, surfactin production

## Abstract

Surfactins are lipopeptide-type biosurfactants produced mainly by *Bacillus* species, consisting of a peptide loop of seven amino acids and a hydrophobic fatty acid chain (C_12_–C_16_). These molecules have been proven to exhibit various biological activities; thus, their therapeutic and environmental applications are considered. Within the surfactin lipopeptide family, there is a wide spectrum of different homologues and isomers; to date, more than 30 variants have been described. Since the newest members of these lipopeptides were described recently, there is no information that is available on their characteristic features, e.g., the dependence of their production from different cultivation parameters. This study examined the effects of both the different carbon sources and various metal ions on the surfactin production of a selected *B. subtilis* strain. Among the applied carbon sources, fructose and xylose had the highest impacts on the ratio of the different variants, regarding both the peptide sequences and the lengths of the fatty acids. Furthermore, the application of metal ions Mn^2+^, Cu^2+^ and Ni^2+^ in the media completely changed the surfactin variant compositions of the fermenting broths leading to the appearance of methyl esterified surfactin forms, and resulted in the appearance of novel surfactin variants with fatty acid chains containing no more than 11 carbon atoms.

## 1. Introduction

**Figure 1:**
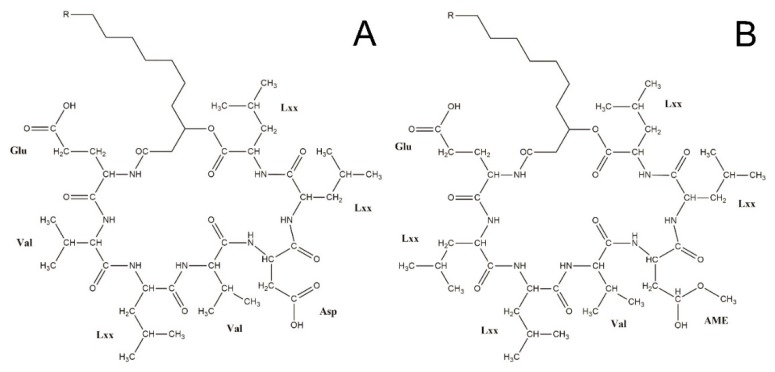
The structure of the surfactin variant [Val2] described by Bóka et al. [18] (**A**) and [AME5] identified by Kecskeméti et al. [19] (**B**). R = C_3_H_7_–C_5_H_11_.

Surfactins are biosurfactants belonging to the family of cyclic lipopeptides, which were first described in 1968 [1], and are mainly produced by gram-positive *Bacillus* species including *B. amyloliquefaciens*, *B. licheniformis*, *B. mojavensis*, *B. pumilus*, and *B. subtilis* [2,3,4,5,6]. These molecules were isolated in the form of needle-shaped white crystals, and denominated according to their outstanding surface activities. These compounds exhibit a wide range of different biological activities, such as anti-mycoplasmic [7], anti-viral [8], anti-tumor [9], and anti-inflammatory activities [10]. Due to their surface effect, the application of surfactins in environmental research is also gaining growing interest [11]. Surfactins consist of a hydrophobic β-hydroxy fatty acid ‘tail’ part and a ‘head’ part of seven amino acids connected by a lactone bridge, which forms the cyclic structure of the molecules [12]. The variability of the length of the fatty acid chain and the amino acid sequence results in the presence of numerous surfactin variants and isoforms. Based on their amino acid sequences, 10 naturally produced variants were summarized in 2003 by Bonnmatin et al. [12], including [Ala4] [13], [Lxx4] ([Leu4], [Ile4]) [14], [Val7], [Ile7] [15], [Ile2,4], [Ile2,4,7] [16], [Val2,7], [Val2,Ile7] and [Ile2,Val7] [17] surfactins. However, recent investigations revealed certain novel groups of surfactin molecules. One of them contains valine ([Val2]) in the second amino acid position (Figure 1A) [18], while the application of a more non-polar HPLC (High Performance Chromatography) elution method revealed another group of molecules with a central aspartic acid methyl ester residue (AME) instead of aspartate, which was reported to appear in the fifth position in all previous variants leading to the discovery of the [AME5], [AME5,Val7], and [Lxx4,AME5] variants (Figure 1B) [19,20,21,22]. Furthermore, it was also discovered that the molecular weight of surfactins could range from 993 Da to 1049 Da, and that the attached fatty acid chain length could be within the range of C13–C18 [18,19].

Different *Bacillus* species and strains could produce distinct variants of these lipopeptides in various ratios. Moreover, in the formation of the surfactin profile, both the cultivation parameters and genetically encoded differences could play an important role. The effects of various cultivation parameters affecting the cellular growth, including pH, temperature, dissolved oxygen concentration, and degree of aeration, on the total surfactin production were also described in detail in the literature [2,23,24,25,26]. Furthermore, several investigations have been performed to elucidate the most favorable media compositions for the highest surfactin yield, which included the examination of complex media [1] supplemented with different nitrogen sources, trace elements, different carbon sources, and precursors of surfactins [2,24,27,28]. Remarkable surfactin production were also achieved in solid state fermentation with the *B. pumilus* UFPEDA 448 strain, using a medium based on okara, with the addition of sugarcane bagasse. On this solid support, the secreted bacterial proteases could pre-hydrolyze the okara proteins, stimulating the lipopeptide biosynthesis [29]. Related to the lipopeptide amounts, it was also observed that isolate *B. licheniformis* W16 showed the highest biosurfactant activities, using either glucose or cane molasses as a carbon source [30].

The four trace elements, Mg^2+^, K^+^, Mn^2+^, and Fe^2+^ are particularly important in the biosynthesis of surfactin in *B. subtilis* [28,29,30,31,32,33,34,35,36,37], while the presence of glutamate could also improve the amount of the produced lipopeptides [38]. A series of carbohydrate-based (glucose, sucrose, galactose, maltose, sucrose, mannitol, soluble starch, and dextrin) media inoculated with *B. subtilis* was also tested, which revealed that sucrose was the optimal carbon source for the highest surfactin yield [39,40,41]. However, despite the extensive efforts to study the influence of various culture conditions on the total surfactin production, there is only limited knowledge about the effects of these parameters on the production of different variants, and their relative amounts in the lipopeptide mixture. The observed basic surfactin profiles of the extracts were only compared to the commercial surfactin, and the alteration of the profiles after the modifications of the media were not followed, just the total yield of surfactins [28,41]. Generally, even if chromatographic separation was applied, the identities of the individual peaks were not further investigated [37]. In certain cases, one of the variants was highlighted and selected to monitor the effects of changed culturing parameters such as C15–[Sur] optimizing the surfactin secretion of *B. amyloliquefaciens* [40]. In the literature, only Akpa et al. [42] reported that the culture conditions could possess an influence on the percentages of the different homologous surfactin variants produced by *B. subtilis* S49. However, this work could focus only on the [Sur] and [Val7] (named as S1 and S2) species discovered until that time, with the fatty acid chain lengths of C13–C17 and C13, respectively [42,43].

In the present study, the originally applied culture medium of our frequently investigated *B. subtilis* strain SZMC 6179J was modified by the application of various carbon sources and metal ions, and the differences in the relative quantity of surfactin variants and isoforms in the fermenting broths were observed and compared. For the purposes of the identification of the surfactin variants and isoforms (Table 1), an HPLC-MS (Mass Spectrometry) method was used, based on our previous work in discovering three new surfactin types by applying HPLC MS/MS measurements [19]. 

## 2. Results and Discussion

### 2.1. Effects of Various Carbon Sources on Surfactin Production 

The chromatographic and mass spectrometric methods reported in our previous studies were able to determine a very large range of already-described variants and also allowed for the identification of novel compounds [18,19,20,21]; therefore, it could be applied to follow the changes of the surfactin profile caused by different influences. Firstly, modified culture media were applied to examine the effect of various carbon sources on the surfactin production of *B. subtilis* SZMC 6179J, and on the qualitative and relative quantitative compositions of the different variants. For these purposes, nine different carbon sources (cellobiose, starch, maltose, ethanol, mannitol, fructose, glycerol, sucrose and xylose) were used besides the original culture conditions reported by Besson et al. [38]. The other culture parameters and the sample preparation were the same in all cases. The relative amounts of the surfactin variants listed in Table 1 were determined based on the integrated area ratio on the extracted ion chromatograms of their sodiated molecular ions after the HPLC-MS analyses (Table 1).

Comparing the EICs (Extracted Ion Chromatogram) of the extracts prepared from the *Bacillus* strain cultivated on the examined carbon sources with the original profile observed on a glucose-based medium revealed that the chromatograms followed the same pattern, and no new variants appeared, due to the modification of the carbon source. However, the intensity of the corresponding peaks, as well as the relative ratios of the different variants within a chromatogram, differed from each other in certain cases, suggesting that the examined *Bacillus* strain produced surfactin variants in different ratios, due to the change of carbon source in the culture media. Altogether, 22 variants were produced, consisting of five [Sur], two [Val2], five [Val7], two [Val2,7], four [AME5], two [AME5,Val7], and two [Lxx4,AME5] homologues (Table 1). If the isobaric fatty acid chains (like the iso, or anteiso chains) or the Leu/Ile residue differences were taken into account, this number could be even higher. The [Sur] variant contained a β-hydroxy fatty acid chain in C13–C17 lengths, and the [Val2] was linked to the C13 and C15 chains, while the fatty acid parts of [Val7], [Val2,7], [AME5], [AME5,Val7], and [Lxx4,AME5] surfactins were in the ranges of C13–C17, C14–C15, C15–C18, C17–C18 and C16–C17, respectively.

**Figure 2:**
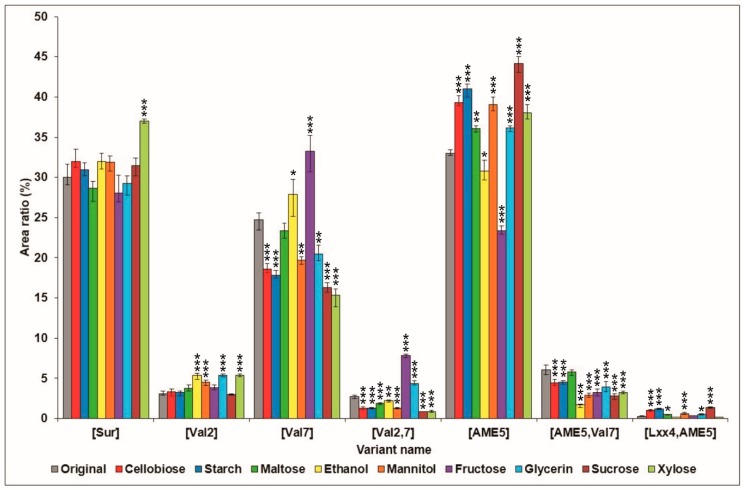
Comparison of relative area of distribution of the surfactin variant groups produced by *B. subtilis* in the original culture medium with the altered carbon sources. Significance levels of the differences from the control are marked with asterisks: *** *p* < 0.001; ** *p* < 0.01; * *p* < 0.05.

To compare the production ratios of the different surfactin variant groups among each of the applied carbon sources, the integrated areas of all the surfactin peaks were calculated, and their percentage ratios are shown in Figure 2, while the ratio data of the whole variants were presented in Supplementary Tables S1–S13.

The largest impact was observed in the cases of fructose and xylose. The application of fructose resulted in a significant decrease in the amount of variants containing AME in the fifth position of their peptide sequence ([AME5], [AME5,Val7]), while a significant increase was observed in the amount of variants containing Val in their seventh position ([Val7], [Val2,7]). Xylose was the sole carbon source, causing a significant effect on the [Sur] amount, positively influencing its production. Beside [Sur], a significant increase was also observed among the [Val2] and [AME5] variants, but the amount of [Val7], [Val2,7], and [AME5,Val7] was reduced, suggesting that the incorporation of Val into the seventh position of the peptide chain was not favored with xylose as the carbon source. Cellobiose, starch, and sucrose applied into the cultivation media showed the same effects on the profile of the surfactin groups, resulting in a decrease of [Val7], [Val2,7] and [AME5,Val7], as well as in the increase of [AME5] and [Lxx4,AME5] secretions, with high (*p* ≤ 0.001) significance. Similarly, the presence of maltose in the media also resulted in higher [AME5] and [Lxx4,AME5] levels, nevertheless, it had no significant negative effects on the relative amounts of [Val7] and [AME5,Val7], only on [Val2,7] (Figure 2). Similarly to xylose, ethanol, mannitol and glycerol in the media could improve and reduce the amounts of [Val2] and [AME5, Val7], respectively, but they had different effects on the production of [Val7], [Val2,7], [AME5], and [Lxx4,AME5].

To summarize our results from the point of view of the surfactin groups, it could be concluded that the modification of the culture medium by different carbon sources did not have significant effects (except for xylose) on the relative production ratio of the firstly described surfactin variant ([Sur]). In the case of the [Val2] molecules, no meaningful effect could be seen on the production ratios; a small increase of about 2% occurred in the samples produced on media supplemented with ethanol, mannitol, glycerol, or xylose. As for the [Val7] surfactins, only the samples from ethanol- and fructose-modified media showed an increase in relative percentage, the former by 3%, the latter by 9%, but cellobiose, starch, sucrose, xylose, mannitol, and glycerol decreased their amounts. Among the other samples, the one with xylose as the carbon source in the culture medium was affected in the most negative way, as it had suffered a decrease of more than 9% of [Val7]. Regarding the ratios of the [Val2,7] variants, the only change worth mentioning could be observed both in the samples from the glycerol- and fructose-modified media, as they grew by more than 1.5% and 5%, being almost double and triple that of the original sample, respectively. In the case of the novel surfactin molecules characterized by Kecskeméti et al. [19], the ratios of the [AME5] variants grew in the presence of cellobiose, starch, maltose, mannitol, glycerol, sucrose, and xylose carbon sources, by 6%, 8%, 3%, 6%, 3%, 11%, and 5%, respectively. Only the application of ethanol and fructose resulted in a decrease, by less than 3% for the former, but by almost 10% for the latter. Modification of the culture media reduced only the ratio of the [AME5, Val7] surfactins, this effect was the most visible in the case of ethanol, where their percentage declined by more than 4%. Most of the carbon sources significantly affected the production of the [Lxx4, AME5] variant, but they had no notable impact, as only the use of starch and sucrose caused its ratio to exceed 1%.

**Figure 3:**
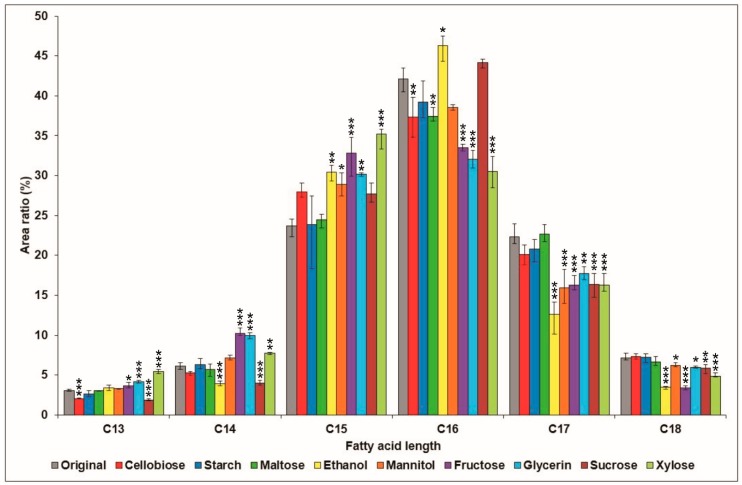
Comparison of the relative area distribution of *B. subtilis* surfactins, grouped by their fatty acid chain lengths in the original culture medium with the altered carbon sources. Significance levels of the differences from the control are marked with asterisks: *** *p* < 0.001; ** *p* < 0.01; * *p* < 0.05.

The ratio of the molecule groups possessing various fatty acid chain lengths was also investigated and compared to the control cultivation medium (Figure 3). The application of starch had no significant effect on the production of these groups, while the supplementation of maltose, cellobiose, and sucrose had only significant negative effects to one (C16), two (C13, C16), and four (C13, C14, C17, C18) groups, respectively. Both the applications of mannitol and ethanol increased the relative amounts of the groups C15 and C15–C16, respectively, but also caused the reduction of the occurrence of certain groups. Furthermore, the same effects could be observed in the case of the media supplemented with fructose, glycerol, and xylose, as the sum production of the groups containing the shorter fatty acid chains (C13–C15) was incremented, while the secretion of molecules with longer fatty acid chains (C16–C18) was reduced significantly.

It was also demonstrated that the effect of the carbon source modification was negligible in the case of the C13 surfactins (Figure 3). The largest, a 2% increase, occurred in the samples containing xylose, while using sucrose decreased the ratio by only slightly more than 1%. The results were similar among the C14 molecules, with the application of fructose causing an increase of 4%, and the use of ethanol decreasing the ratio by about 2%. However, four carbon sources increased the relative percentage of the C15 variants significantly, with xylose being the most efficient, with a rise of more than 11%. The greatest impact of the utilization of different carbon sources can be seen in the compounds possessing a fatty acid chain of 16 carbon atoms. While using ethanol resulted in a growth of 4%, cellobiose, maltose, fructose glycerol, and xylose affected the ratios in a diminishing way, with the latter three carbon sources decreasing them by 8%, 10%, and 11%, respectively. Declines were also observed in the case of both C17 and C18 surfactins, but the area ratio of the longest surfactin variants yet identified was relatively unaffected by these modifications (Figure 3).

### 2.2. Effects of Culture Media Supplemented with Various Metal Ions on Surfactin Production

To examine the effects of the culture medium modification on the production of surfactins, HPLC-MS analyses were conducted on the fermenting broths of *B. subtilis* cultivated with the supplementation of manganese, copper, and nickel ions. The resulting surfactin patterns of these samples were, however, different from those of both the original and the carbon source-modified cultivation parameters, due to the appearance of new peaks. These newly detected peaks could not be identified based on the original sample, therefore MS^2^ analyses were required by applying an ion-trap mass analyzer. 

**Figure 4:**
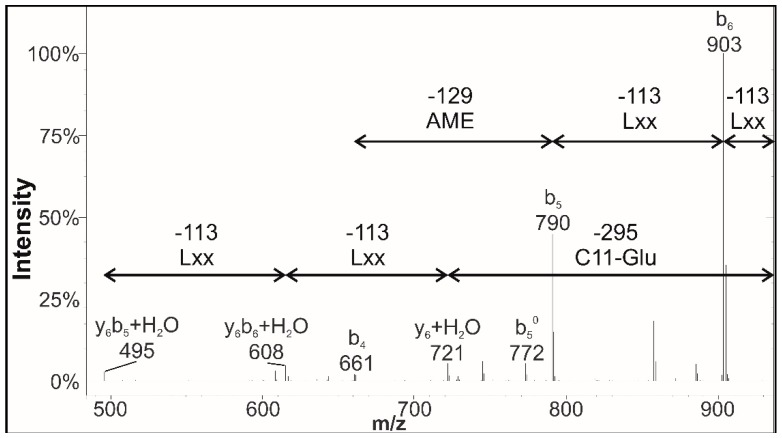
The MS^2^ spectrum of a peak at Rt = 76.89 min (*m*/*z* = 1016) of the fermenting broth of *B. subtilis* cultivated in culture medium supplemented with manganese.

The MS^2^ spectrum of a peak at the *m*/*z* value of 1016 of the sample from the medium supplemented with manganese is shown as an example (Figure 4). On the MS^2^ spectra of the surfactin variants, more fragmented ion series could be recognized. The first member of these formed by the internal fragmentation mechanism (y_6_ + H_2_O) [14] possessed the same mass difference of 14 Da as that characterized by Kecskeméti et al. [19], suggesting that the surfactin molecule contains AME in the fifth amino acid position. The following fragment ions of this series (y_6_b_6_ + H_2_O, y_6_b_5_ + H_2_O) suggested that both the seventh and sixth residues of the peptide sequence are Lxx residues. The mass differences between the fragments of the simple cleavage mechanism (b_6_, b_5_, and b_4_) [44] also confirms that the last three amino acids at the C-terminal are Lxx, Lxx, and Glu. Furthermore, the *m/z* difference between the sodiated precursor ion and the first internal fragment (y_6_ + H_2_O) indicates that the fatty acid chain length is C11, as it should be in the case of a [AME5] variant with the *m/z* value of 1016; a surfactin isoform having such short aliphatic part has not been reported before (Figure 4). Compared to the samples from the original medium and the carbon source-modified media, 10 new molecules were found in the fermenting broth of *B. subtilis* cultivated with the addition of metal ions, including C12–[Sur], C14–[Val2], C11–C12–[AME5], C12–C16–[AME5,Val7], and C15–[Lxx4,AME5]. Further examinations of the MS^2^ spectra of these samples revealed that the variant possessing a central AME residue appeared in all samples deriving from media supplemented with metal ions. 

In the previous examinations, Cooper et al. reported that the addition of manganese and iron salts enhanced the yield of surfactin [31]. The increase in surfactin production due to iron-enriched culture media has been studied extensively by Wei et al. [33,34,35]. Firstly, it was observed that 2–4 mM iron either initially or during batch culture and keeping the pH above 5.0 could improve the total amount of surfactin regarding to the sum concentration of the six most abundant peaks [33]. Furthermore, the applied iron supplement strategies were optimized following a growth-associated kinetic model reaching a higher level of the total surfactin (162 mg/g dry cell) [35]. It has been also reported that the major role of the metal ions there is in the alteration of nitrogen metabolism, leading to improved lipopeptide synthesis. In continuous-phased growth of *B. subtilis*, the increase of the manganese reduced the requirement for nitrogen resulting from high surfactin production [32]. Furthermore, change of nitrogen and K^+^ uptake resulted in the promotion of surfactin synthesis [28,36]. In conclusion, the different metal ion supplementations in the fermentation media could influence the yield of the surfactins significantly, via complex processes involving cell growth intensification, affected nitrogen utilization, and other possible yet unknown mechanisms [45].

Evidence for the essential role of metal ions on the biosurfactant production of these microorganisms was revealed previously. However, in those studies, comparisons of the total surfactin yield were only performed, while the relative amounts of the different lipopeptide variants and the changes of their ratios due to the addition of various metal ions is not known yet.

**Figure 5:**
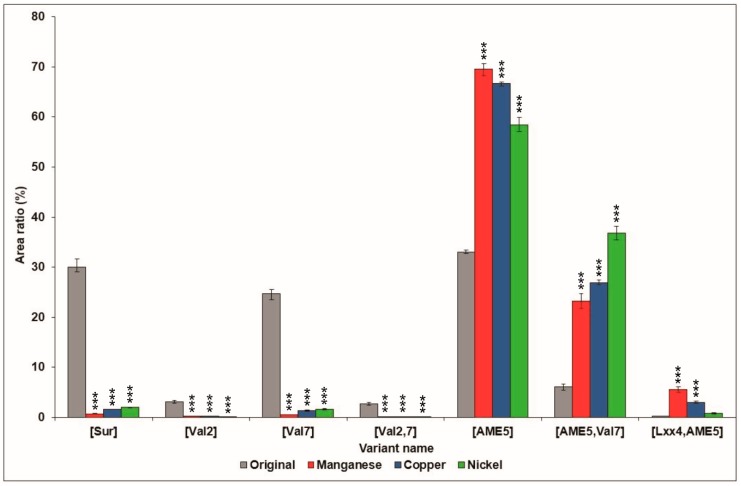
Comparison of relative area distribution of surfactin variants produced by *B. subtilis* in the original culture medium, and the media supplemented with manganese, copper, and nickel. Significance levels of the differences from the control are marked with asterisks.

For the observation of the relative amounts of the different surfactin molecules, the integrated peak areas of the various isoforms in the sample from the original medium were compared with the ones derived from the media modified with the different metal ions (Figure 5). It could be concluded that the presence of the recently characterized variants was dominating, being over 96% in all three extracts, while the relative amount of the [Sur], [Val2], [Val7], and [Val2,7] surfactins was almost negligible, and neither of them reached 2% in any of the modified samples. This suggests that the addition of metal ions may selectively affect the amino acid sequences of the compounds to the AME5 residue in the fifth position, such as [AME5], [AME5,Val7], and [Lxx4,AME5] produced by the bacterium.

**Figure 6:**
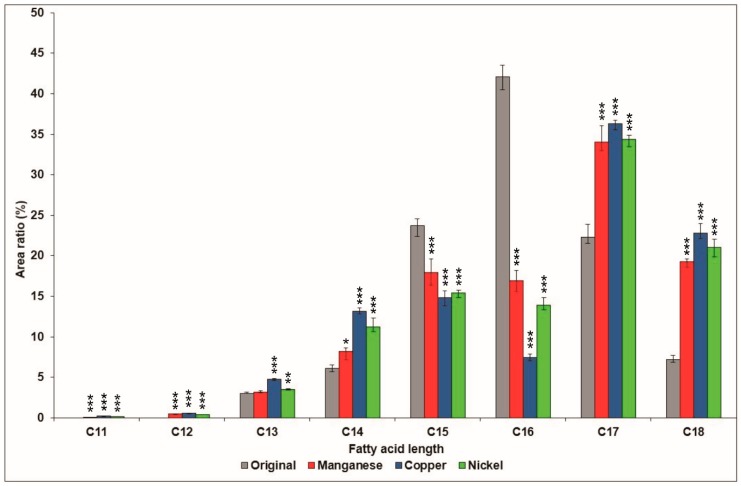
Comparison of the relative area of distribution of fatty acid chain lengths of surfactins produced by *B. subtilis* in the original culture medium and in the media supplemented with manganese, copper, and nickel. Significance levels of the differences from the control are marked with asterisks: *** *p* < 0.001; ** *p* < 0.01; * *p* < 0.05.

The ratios of the different fatty acid chain lengths were also examined within surfactin population, comparing the extracts of the applied strain growing on media supplemented with different metal ions (Figure 6). The relative amount of the newly detected C11 and C12 surfactin homologues were rather low, neither of them reached 1%, while in the case of the C13 and C14 molecules, a slight increase could be observed in comparison with the original sample; with the extract from the copper-modified medium presenting the largest increases in the relative amounts of both homologues (2% and 7%, respectively). However, a notable decrease in the overall percentage could be observed in the case of the C15 and C16 variants, appearing between 14–18% and 7–17% in the samples from the supplemented media, while the amount in the samples from the original medium was approximately two times higher. Originally, these two chain lengths were the most dominant, but thanks to the application of metal ions in the culture media, the relative amounts of both C17 and C18 exceeded them. Examining these last two fatty acid chain lengths, a remarkable escalation in their relative percentages could be observed, reaching up to a 12–15% increase in both cases. These two homologues represented more than 50% of the detected variants in the extracts from all three metal ion-modified media; furthermore, more than two thirds of the surfactins possessed aliphatic chain lengths above 15 carbon atoms.

## 3. Materials and Methods 

### 3.1. Chemicals and Reagents

All solvents and reagents were analytical or of the highest grade available. Hydrochloric acid, methanol, and acetonitrile were purchased from VWR (Budapest, Hungary). Trifluoroacetic acid (TFA) was purchased from Sigma Aldrich (Budapest, Hungary). HPLC-grade water was produced by ultrafiltration with a Millipore Milli-Q Gradient A10 water purification system (Merck, Budapest, Hungary).

### 3.2. Microorganism and Culture Conditions

The examined *B. subtilis* strain SZMC 6179J (GenBank Accession numbers: JX683908.1, gyrA; NZ_CP015004.1, complete genome), isolated from the tomato rhizosphere by Vágvölgyi et al. [46] and possessing high-level antagonistic properties against plant pathogens, was previously examined as the producer of novel surfactins [18,19]. The strain was derived from the Szeged Microbiology Collection (SZMC; www.szmc.hu), and it was maintained on nutrient agar (5 g/L peptone, 3 g/L yeast extract, 5 g/L NaCl, 15 g/L agar) slants, and stored at 4 °C.

For surfactin production, a liquid ferment broth was applied according to Besson et al. [38] containing 10 g/L glucose, 5 g/L glutamic acid, 1 g/L KH_2_PO_4_, 1 g/L K_2_HPO_4_, 1 g/L KCl, 500 mg/L MgSO_4_·7H_2_O, 5 mg/L FeSO_4_·7H_2_O and 160 µg/L CuSO_4_·5H_2_O. Bacteria (5 × 10^7^ cells) were inoculated into 50 mL medium in 250 mL Erlenmeyer flasks, followed by the incubation on a rotary shaker at 120 rpm for 5 days at 25 °C. For the modification of the cultivation parameters by carbon sources, glucose was changed separately to cellobiose, ethanol, starch, maltose, mannitol, fructose, sucrose, glycerol, or d-xylose. To test the effects of metal ions, the culture medium was supplemented separately with 1 mM final concentrations of manganese, copper, or nickel ions from the stock solution of MnSO_4_·H_2_O, CuSO_4_·5H_2_O and NiSO_4_·7H_2_O metal salts, respectively. The effect of each parameter was tested in three replicate experiments. 

### 3.3. Extraction and Measurement of Surfactin Variants

After cultivation, the fermentation materials were centrifuged at 8000× *g* for 10 min to separate the bacterial cells from the ferment broths. The supernatants were then recovered, and their pH was set to 2 with 1 M HCl solution and incubated overnight at 4 °C to precipitate the lipopeptides. The pellets were collected by centrifugation (10,000× *g*, 10 min) and resolved in 3 mL methanol [18]. Five microliters of each extract was injected into the HPLC-MS system (Shimadzu, Germany) consisting of a pump (LC-10ADVP), a degasser (DGU-14A), a column thermostat (CTO-10ASVP), an autosampler (SIL-10ADVP), a system controller (SIL-10ADVP), and a single quadrupole MS (LCMS-2010A). The columns were thermostated at 35 °C using a model 7990 Space column heater (Jones Chromatography, Hengoed, UK). Gradient RP-HPLC elution was carried out on a Phenomenex Prodigy analytical column (100 × 2.0 mm, 3 µm; Gen-Lab, Budapest, Hungary), while the applied gradient contained two eluents including H_2_O (A) and a mixture of acetonitrile:methanol (1:1, *v*/*v*, B) supplemented each with 0.05% TFA. The gradient elution was applied at a flow rate of 250 µL/min, and it was started with 47% of eluent B for 12 min and increased linearly to 95% at 92 min, with the value being held for 16 min then decreased to the initial 15% in two minutes and kept constant for 10 min, resulting in a run of 120 min in total. 

For the MS, an ESI source was applied in positive mode monitoring selected ions (SIM) with the following MS parameters: an ESI interface voltage 1.5 kV, CDL temperature 250 °C, and a block heater temperature 200 °C. Nitrogen was the nebulizing gas, with a flow rate of 1.5 L/min. The drying gas pressure was set to 0.1 MPa. The SIM ions were *m*/*z* 1016, 1030, 1044, 1058, 1072, 1086, 1100, 1114, and 1128. The qualitative analysis of the known surfactin variants were based on our previous work, which dealt with the identification and structural elucidation of surfactin variants by ion trap analyzer using the retention time (elution order) and sodiated molecular ion *m/z* value data.

### 3.4. Structural Elucidation of Novel Surfactins

Previously undetected surfactin homologues extracted from the *B. subtilis* strain SZMC 6179J were identified by HPLC-ESI-IT-MS with an Agilent 1100 Series HPLC system (Palo Alto, CA, USA) equipped with a binary pump, a vacuum degasser, and a µWell-plate autosampler. The chromatographic parameters were the same as mentioned above at a single quadrupole system, while the injection volume was 3 µL.

The MS^2^ analyses were performed on a 500-MS ion trap mass spectrometer (Agilent, Palo Alto, CA, USA) equipped with an ESI source in positive mode based on our previous study [19]. The utilized ESI parameters were: spray chamber temperature, 50 °C; drying gas (N_2_) pressure and temperature, 30 psi and 350 °C, respectively; nebulizer gas (N_2_) pressure, 50 psi; needle voltage, 5000 V; spray shield voltage, 600 V. The general parameters were: maximum scan times, 1.51 s/scan; scans averaged, 2 µscans; data rate, 0.66 Hz; multiplier offset, 0 V. Ionization control parameters were: target TIC, 100%; max ion time, 250,000 µs. The MS^2^ measurements were achieved using the same ESI source and general MS parameters mentioned above. For the unknown variants, the 1016, 1030, 1044, 1058, 1072, 1086, 1100, 1114, and 1128 *m*/*z* values were monitored and the following excitation storage level (*m*/*z*) /excitation amplitude values were applied: (V) 273.5/3.91, 277.4/9.96, 281.3/4.02, 285.3/4.07, 289.3/4.12, 293.3/4.17, 297.4/4.22, 301.5/4.27, and 305.6/4.32, respectively. Each *m/z* value of the sodium adducts was monitored alone in separated chromatographic runs, to avoid the possible cross-talk effects in the mass analyzer. 

### 3.5. Nomenclature

Surfactin isoforms were designated according to Grangemard et al. [16], Bóka et al. [18], and Kecskeméti et al. [19]. The esterified forms of aspartic acid at the side chain carboxyl group were abbreviated as AME, and as the applied mass spectrometric technique could not distinguish between the Leu and Ile isobaric residues, this sequence element was usually marked as Lxx.

The first discovered surfactin sequence (Glu-Lxx-Lxx-Val-Asp-Lxx-Lxx) was marked as [Sur], and any alterations in the peptide sequence were indicated with the abbreviation and position of the changed amino acid, such as [Val2] (Glu-Val-Lxx-Val-Asp-Lxx-Lxx), [Val2] (Glu-Lxx-Lxx-Val-Asp-Lxx-Val), [Val2,7] (Glu-Val-Lxx-Val-Asp-Lxx-Val), [AME5] (Glu-Lxx-Lxx-Val-AME-Lxx-Lxx), [AME5, Val7] (Glu-Lxx-Lxx-Val-AME-Lxx-Val), and [Lxx4,AME5] (Glu-Lxx-Lxx-Lxx-AME-Lxx-Lxx). Furthermore, the fragment ions on the MS^2^ spectra were designated according to the terminology published by Roepstorff and Fohlman [47], as well as Biemann [48], while the internal fragments of sodiated fragment ions were designated by the y_n_b_m_ nomenclature [18,49].

### 3.6. Statistical Analysis

All the statistical analyses were performed using GraphPad Prism version 7.0 for Windows (GraphPad Software, San Diego, CA, USA, 2016). The significant differences between the amount of surfactin variants produced in different culturing environments and the control fermentations were determined by one-way analysis of variance with Dunett's multiple comparison post-test.

## 4. Conclusions

Based on our work, it could be concluded that the culturing conditions could significantly influence the relative amounts of the individual members of surfactins produced by the selected *B. subtilis* strain, which seems to be selective in certain cases for some variants. Furthermore, the supplementation of trace elements (manganese, copper and nickel) could lead to the emergence of the production of new surfactin homologues. However, it is important to consider that the mentioned modifications may have different effects on other *B. subtilis* strains, possibly promoting the forming of other surfactin profiles, and even the production of other novel variants.

The cultivation parameters of *Bacillus subtilis* strain SZMC 6179J were modified by changing the carbon source of the culture medium from glucose to cellobiose, ethanol, starch, maltose, mannitol, fructose, sucrose, glycerol, or xylose, as well as by supplementing the original culture medium with manganese, copper, or nickel ions, to examine the differences regarding the production of surfactin molecules, and to compare their relative amounts to the ones in the sample from the original glucose medium. For these purposes, an HPLC-MS method was applied, which is capable of measuring all currently described surfactin variants, including the recently discovered AME-modified ones ([AME5], [AME5,Val7], and [Lxx4,AME5]) characterized in our previous study. The detected surfactins were examined by their relative ratios of different variants, grouped by both their peptide sequence and the various chain lengths of the homologues. It was found that the use of carbon sources other than glucose had effects on the ratios of the produced surfactins, showing selectivity in certain cases, while the application of metal ions had a larger impact and enabled the discovery of surfactin variants possessing AME5 in their fifth amino acid position, which was the most dominant group in the extracted fermenting broth from the metal ion-supplemented media. Furthermore, these metal ions promoted the production of compounds having longer fatty acid chains: two thirds of the detected molecules were C16, C17, or C18 homologues. The application of these ions therefore has the potential for selectively producing a group of surfactins to examine their possible specific biological activities. 

## Figures and Tables

**Table 1:** List of the initially examined surfactins produced by *B. subtilis* SZMC 6179J.

Molecular Mass	Fatty Acid Chain	Peptide Sequence ^1^
993	C13	[Val2], [Val7]
	C14	[Val2,7]
1007	C13	[Sur]
	C14	[Val7]
	C15	[Val2,7]
1021	C14	[Sur]
	C15	[Val2], [Val7]
1035	C15	[Sur]
	C16	[Val7]
1049	C15	[AME5]
	C16	[Sur]
	C17	[Val7]
1063	C16	[AME5]
	C17	[AME5,Val7], [Sur]
1077	C17	[AME5]
	C18	[AME5,Val7]
1091	C17	[Lxx4,AME5]
	C18	[AME5]
1105	C18	[Lxx4,AME5]

^1^ For the peptide sequences, see the Materials and Methods section.

## Supplementary Materials

The following are available online.
