# Supplementary material for: Effects of Different Cultivation Parameters on the Production of Surfactin Variants by a Bacillus subtilis Strain
Source: Molecules. 2018 Oct 18;23(10):2675. doi: 10.3390/molecules23102675 (PMC6222309; doi:10.3390/molecules23102675)
Supplement: Supplementary file 1 [file molecules-23-02675-s001.pdf]

Table S1. Percentage ratios of the different surfactin variants and homologues in the original sample.

Original							
**Ratio of variant and deviation (%)**							
	[Sur]	[Val2]	[Val7]	[Val2,7]	[AME5]	[AME5,Val7]	[Lxx4,AME5]
C13	1.62 (0.07)	0.20 (0.01)	1.09 (0.06)	nd.	nd.	nd.	nd.
C14	3.39 (0.10)	nd.	2.10 (0.17)	0.37 (0.03)	nd.	nd.	nd.
C15	6.67 (0.30)	2.92 (0.27)	4.15 (0.34)	2.38 (0.20)	6.58 (0.14)	nd.	nd.
C16	11.42 (0.86)	nd.	13.68 (0.84)	nd.	15.02 (0.26)	nd.	0.18 (0.02)
C17	6.90 (0.33)	nd.	3.69 (0.41)	nd.	7.85 (0.59)	2.80 (0.31)	0.12 (0.01)
C18	nd.	nd.	nd.	nd.	3.58 (0.06)	3.29 (0.31)	nd.

nd. – not detected

Table S2. Percentage ratios of the different [Sur]factin variants and homologues in the sample modified with cellobiose carbon source.

Cellobiose							
**Ratio of variant and deviation (%)**							
	[Sur]	[Val2]	[Val7]	[Val2,7]	[AME5]	[AME5,Val7]	[Lxx4,AME5]
C13	1.17 (0.07)	0.20 (0.01)	0.68 (0.04)	nd.	nd.	nd.	nd.
C14	3.95 (0.35)	nd.	1.16 (0.07)	0.13 (0.02)	nd.	nd.	nd.
C15	10.43 (0.95)	3.17 (0.45)	2.68 (0.17)	1.17 (0.22)	10.52 (0.58)	nd.	nd.
C16	9.25 (0.56)	nd.	11.82 (0.51)	nd.	15.55 (1.58)	nd.	0.71 (0.09)
C17	7.19 (0.73)	nd.	2.22 (0.14)	nd.	8.78 (0.62)	1.64 (0.20)	0.28 (0.02)
C18	nd.	nd.	nd.	nd.	4.49 (0.47)	2.81 (0.18)	nd.

nd. – not detected

Table S3. Percentage ratios of the different [Sur]factin variants and homologues in the sample modified with starch carbon source.

Starch							
**Ratio of variant and deviation (%)**							
	[Sur]	[Val2]	[Val7]	[Val2,7]	[AME5]	[AME5,Val7]	[Lxx4,AME5]
C13	1.61 (0.20)	0.23 (0.01)	0.65 (0.02)	nd.	nd.	nd.	nd.
C14	4.58 (0.28)	nd.	1.28 (0.17)	0.13 (0.01)	nd.	nd.	nd.
C15	8.89 (0.70)	2.98 (0.31)	2.71 (0.19)	1.18 (0.10)	10.96 (0.46)	nd.	nd.
C16	8.72 (0.72)	nd.	10.62 (0.10)	nd.	17.27 (0.67)	nd.	0.73 (0.04)
C17	7.12 (0.65)	nd.	2.59 (0.23)	nd.	8.82 (0.82)	1.63 (0.08)	0.42 (0.07)
C18	nd.	nd.	nd.	nd.	3.98 (0.35)	2.88 (0.15)	nd.

nd. – not detected

Table S4. Percentage ratios of the different [Sur]factin variants and homologues in the sample modified with maltose carbon source.

Maltose							
**Ratio of variant and deviation (%)**							
	[Sur]	[Val2]	[Val7]	[Val2,7]	[AME5]	[AME5,Val7]	[Lxx4,AME5]
C13	1.56 (0.04)	0.25 (0.01)	1.18 (0.08)	nd.	nd.	nd.	nd.
C14	3.35 (0.36)	nd.	1.72 (0.49)	0.27 (0.02)	nd.	nd.	nd.
C15	7.07 (0.39)	3.39 (0.43)	3.56 (0.11)	1.49 (0.14)	8.39 (0.56)	nd.	nd.
C16	9.35 (0.25)	nd.	13.28 (0.05)	nd.	15.22 (1.12)	nd.	0.17 (0.02)
C17	7.28 (1.53)	nd.	3.90 (0.05)	nd.	9.05 (0.83)	2.86 (0.01)	0.32 (0.03)
C18	nd.	nd.	nd.	nd.	3.67 (0.36)	2.66 (0.26)	nd.

nd. – not detected

Table S5. Percentage ratios of the different [Sur]factin variants and homologues in the sample modified with ethanol carbon source.

Ethanol							
**Ratio of variant and deviation (%)**							
	[Sur]	[Val2]	[Val7]	[Val2,7]	[AME5]	[AME5,Val7]	[Lxx4,AME5]
C13	1.72 (0.17)	0.33 (0.02)	1.26 (0.07)	nd.	nd.	nd.	nd.
C14	2.12 (0.37)	nd.	1.52 (0.13)	0.22 (0.03)	nd.	nd.	nd.
C15	7.04 (0.36)	4.93 (0.35)	3.70 (0.11)	1.95 (0.17)	12.34 (0.54)	nd.	nd.
C16	12.72 (0.48)	nd.	18.43 (0.57)	nd.	14.42 (0.94)	nd.	0.01 (0.00)
C17	7.91 (0.12)	nd.	4.03 (0.48)	nd.	0.24 (0.01)	1.60 (0.22)	0.14 (0.01)
C18	nd.	nd.	nd.	nd.	3.33 (0.21)	0.06 (0.01)	nd.

nd. – not detected

Table S6. Percentage ratios of the different [Sur]factin variants and homologues in the sample modified with mannitol carbon source.

Mannitol							
**Ratio of variant and deviation (%)**							
	[Sur]	[Val2]	[Val7]	[Val2,7]	[AME5]	[AME5,Val7]	[Lxx4,AME5]
C13	1.95 (0.05)	0.37 (0.05)	0.96 (0.08)	nd.	nd.	nd.	nd.
C14	5.08 (0.15)	nd.	1.87 (0.32)	0.20 (0.04)	nd.	nd.	nd.
C15	9.86 (0.85)	4.16 (0.45)	2.51 (0.25)	1.09 (0.06)	11.27 (0.40)	nd.	nd.
C16	9.04 (0.88)	nd.	11.96 (0.31)	nd.	17.16 (0.22)	nd.	0.41 (0.08)
C17	5.95 (0.71)	nd.	2.39 (0.34)	nd.	6.52 (1.07)	0.83 (0.19)	0.22 (0.06)
C18	nd.	nd.	nd.	nd.	4.10 (0.09)	2.10 (0.19)	nd.

nd. – not detected

Table S7. Percentage ratios of the different [Sur]factin variants and homologues in the sample modified with fructose carbon source.

Fructose							
Ratio of variant and deviation (%)							
	[Sur]	[Val2]	[Val7]	[Val2,7]	[AME5]	[AME5,Val7]	[Lxx4,AME5]
C13	1.27 (0.08)	0.06 (0.01)	2.22 (0.13)	nd.	nd.	nd.	nd.
C14	3.87 (0.16)	nd.	4.34 (0.15)	1.74 (0.31)	nd.	nd.	nd.
C15	6.51 (0.18)	3.71 (0.16)	10.76 (0.67)	5.95 (0.13)	7.90 (0.59)	nd.	nd.
C16	9.23 (1.14)	nd.	14.78 (0.45)	nd.	8.34 (1.21)	nd.	0.20 (0.02)
C17	6.49 (0.62)	nd.	2.71 (0.32)	nd.	5.08 (0.09)	1.40 (0.14)	0.14 (0.01)
C18	nd.	nd.	nd.	nd.	1.50 (0.23)	1.80 (0.16)	nd.

nd. – not detected

Table S8. Percentage ratios of the different [Sur]factin variants and homologues in the sample modified with glycerin carbon source.

Glycerin							
Ratio of variant and deviation (%)							
	[Sur]	[Val2]	[Val7]	[Val2,7]	[AME5]	[AME5,Val7]	[Lxx4,AME5]
C13	1.20 (0.09)	0.52 (0.04)	2.48 (0.13)	nd.	nd.	nd.	nd.
C14	5.14 (0.18)	nd.	3.97 0.54)	0.81 (0.08)	nd.	nd.	nd.
C15	7.03 (0.41)	4.88 (0.18)	3.31 (0.14)	3.55 (0.28)	11.40 (0.13)	nd.	nd.
C16	9.51 (0.63)	nd.	7.91 (0.23)	nd.	14.33 (0.66)	nd.	0.30 (0.05)
C17	6.32 (0.56)	nd.	2.77 (0.51)	nd.	7.37 (0.65)	1.06 (0.22)	0.18 (0.03)
C18	nd.	nd.	nd.	nd.	3.07 (0.41)	2.89 (0.42)	nd.

nd. – not detected

Table S9. Percentage ratios of the different [Sur]factin variants and homologues in the sample modified with sucrose carbon source.

Sucrose							
**Ratio of variant and deviation (%)**							
	[Sur]	[Val2]	[Val7]	[Val2,7]	[AME5]	[AME5,Val7]	[Lxx4,AME5]
C13	1.03 (0.19)	0.23 (0.00)	0.58 (0.00)	nd.	nd.	nd.	nd.
C14	2.92 (0.22)	nd.	0.99 (0.02)	0.14 (0.01)	nd.	nd.	nd.
C15	9.33 (0.35)	2.81 (0.09)	1.94 (0.08)	0.69 (0.02)	12.16 (0.06)	nd.	nd.
C16	11.77 (0.96)	nd.	11.40 (0.42)	nd.	20.28 (0.21)	nd.	0.83 (0.08)
C17	6.10 (0.48)	nd.	1.19 (0.18)	nd.	8.96 (0.41)	0.60 (0.05)	0.50 (0.03)
C18	nd.	nd.	nd.	nd.	3.53 (0.24)	2.01 (0.38)	nd.

nd. – not detected

Table S10. Percentage ratios of the different [Sur]factin variants and homologues in the sample modified with xylose carbon source.

Xylose							
**Ratio of variant and deviation (%)**							
	[Sur]	[Val2]	[Val7]	[Val2,7]	[AME5]	[AME5,Val7]	[Lxx4,AME5]
C13	3.24 (0.24)	0.91 (0.07)	1.26 (0.07)	nd.	nd.	nd.	nd.
C14	5.89 (0.38)	nd.	1.57 (0.24)	0.28 (0.04)	nd.	nd.	nd.
C15	13.86 (1.17)	4.46 (0.15)	2.07 (0.18)	0.61 (0.09)	14.21 (2.31)	nd.	nd.
C16	6.70 (0.55)	nd.	8.05 (0.92)	nd.	15.68 (1.66)	nd.	0.07 (0.01)
C17	7.31 (0.67)	nd.	2.40 (0.12)	nd.	5.16 (0.48)	1.35 (0.05)	0.08 (0.01)
C18	nd.	nd.	nd.	nd.	2.93 (0.30)	1.91 (0.10)	nd.

nd. – not detected

Table S11. Percentage ratios of the different [Sur]factin variants and homologues in the sample modified with manganese metal ion.

**Manganese**							
**Ratio of variant and deviation (%)**							
	[Sur]	[Val2]	[Val7]	[Val2,7]	[AME5]	[AME5,Val7]	[Lxx4,AME5]
C11	nd.	nd.	nd.	nd.	0.19 (0.00)	nd.	nd.
C12	0.17 (0.01)	nd.	nd.	nd.	0.75 (0.04)	0.17 (0.01)	nd.
C13	0.12 (0.02)	nd.	0.44 (0.04)	nd.	4.49 (0.21)	1.69 (0.28)	nd.
C14	0.90 (0.14)	0.64 (0.07)	0.62 (0.06)	0.19 (0.02)	12.54 (1.05)	2.74 (0.16)	nd.
C15	nd.	nd.	nd.	nd.	28.21 (2.18)	8.81 (0.88)	1.16 (0.17)
C16	0.31 (0.05)	nd.	nd.	nd.	21.69 (3.40)	6.78 (0.24)	7.40 (0.89)
C17	nd.	nd.	nd.	nd.	59.09 (5.68)	10.29 (0.82)	3.28 (0.55)
C18	nd.	nd.	nd.	nd.	21.85 (1.69)	19.18 (1.41)	nd.

nd. – not detected

Table S12. Percentage ratios of the different [Sur]factin variants and homologues in the sample modified with copper metal ion.

**Copper**							
**Ratio of variant and deviation (%)**							
	[Sur]	[Val2]	[Val7]	[Val2,7]	[AME5]	[AME5,Val7]	[Lxx4,AME5]
C11	nd.	nd.	nd.	nd.	0.19 (0.02)	nd.	nd.
C12	0.20 (0.00)	nd.	nd.	nd.	0.12 (0.01)	0.20 (0.02)	nd.
C13	0.18 (0.01)	nd.	0.59 (0.07)	nd.	2.33 (0.05)	1.64 (0.19)	nd.
C14	0.75 (0.02)	0.23 (0.03)	0.77 (0.09)	0.10 (0.01)	6.03 (1.08)	5.38 (0.76)	nd.
C15	nd.	nd.	nd.	nd.	10.70 (0.81)	3.37 (0.19)	0.73 (0.02)
C16	0.48 (0.06)	nd.	nd.	nd.	4.90 (0.36)	0.46 (0.08)	1.62 (0.17)
C17	nd.	nd.	nd.	nd.	28.63 (1.09)	6.94 (0.54)	0.69 (0.06)
C18	nd.	nd.	nd.	nd.	13.77 (0.54)	9.00 (0.54)	nd.

nd. – not detected

Table S13. Percentage ratios of the different [Sur]factin variants and homologues in the sample modified with nickel metal ion.

**Nickel**							
**Ratio of variant and deviation (%)**							
	[Sur]	[Val2]	[Val7]	[Val2,7]	[AME5]	[AME5,Val7]	[Lxx4,AME5]
C11	nd.	nd.	nd.	nd.	0.15 (0.01)	nd.	nd.
C12	0.17 (0.02)	nd.	nd.	nd.	0.10 (0.01)	0.14 (0.02)	nd.
C13	0.14 (0.01)	nd.	0.60 (0.04)	nd.	1.50 (0.06)	1.25 (0.09)	nd.
C14	0.56 (0.02)	0.08 (0.01)	1.04 (0.10)	0.07 (0.02)	4.13 (0.29)	5.31 (0.72)	nd.
C15	nd.	nd.	nd.	nd.	8.21 (0.49)	6.92 (0.16)	0.19 (0.03)
C16	1.11 (0.13)	nd.	nd.	nd.	11.72 (0.42)	0.97 (0.22)	0.45 (0.06)
C17	nd.	nd.	nd.	nd.	23.22 (0.82)	10.81 (1.41)	0.23 (0.05)
C18	nd.	nd.	nd.	nd.	9.46 (1.22)	11.48 (0.09)	nd.

nd. – not detected
